# Diagnostic value of geriatric nutritional risk index and phase angle for malnutrition in patients with COPD: a retrospective cohort study

**DOI:** 10.3389/fnut.2026.1887430

**Published:** 2026-08-19

**Authors:** Xinxin Li, Yaying Yu, Lijing Cao, Shuangjian Huang, Kaiyue Fu

**Affiliations:** 1 Department of Clinical Nutrition, The First Affiliated Hospital of Henan University, Kaifeng, Henan, China; 2 Department of Respiratory and Critical Care Medicine, The First Affiliated Hospital of Henan University, Kaifeng, Henan, China; 3 Department of Public Health, Hebi City Center for Disease Control and Prevention, Hebi, Henan, China

**Keywords:** chronic obstructive pulmonary disease, geriatric nutritional risk index, global leadership initiative on malnutrition, malnutrition, phase angle, receiver operating characteristic

## Abstract

**Background:**

The incidence of malnutrition is relatively high among patients with chronic obstructive pulmonary disease (COPD). The main objective of this study was to explore and compare the discriminative ability of phase angle (PhA) and geriatric nutritional risk index (GNRI) tools for assessing malnutrition among hospitalized patients with COPD who underwent BIA assessment and had available serum albumin data based on the GLIM criteria.

**Methods:**

This single-center retrospective study included 121 patients with hospitalized COPD with BIA measurement and valid serum albumin. All patients underwent body composition analysis and biochemical measurements. Based on the global leadership initiative on malnutrition (GLIM) criteria, patients were categorized into COPD patients with malnutrition and COPD patients without malnutrition. Bootstrap resampling with 1,000 iterations and Hosmer-Lemeshow test evaluate the stability and reliability of the two tools and malnutrition in COPD. More importantly, the exploratory diagnostic efficacies and net clinical benefit of PhA and GNRI tools were assessed for identifying COPD patients with malnutrition using ROC, DeLong test, Decision-curve analysis (DCA).

**Results:**

Using the GLIM malnutrition criteria as the reference, a diagnostic efficacy analysis was conducted using the preset cutoff values of PhA = 4.25° and GNRI = 98. The sensitivity of PhA was 0.691, the specificity was 0.625, the accuracy was 0.669, and the K value was 0.298. The sensitivity, specificity, accuracy and K value were 0.753, 0.700, 0.736 and 0.431 for GNRI. The ROC results showed that the AUC of PhA and GNRI were 0.656 (95% CI: 0.543 ~ 0.769) and 0.785 (95% CI: 0.699 ~ 0.870), respectively. The DeLong test indicated that there was a statistically significant difference in the AUC between the two tools (*p* = 0.035). DCA showed that net clinical benefits of the two tools were similar at low thresholds. As the threshold increased, the net clinical benefit of PhA decreased rapidly and turned negative, while GNRI maintained a positive net clinical benefit in a wider threshold range.

**Conclusion:**

GNRI and PhA showed differing discriminative profiles for GLIM-defined malnutrition. The valid threshold range differed between the two tools. GNRI yielded net clinical benefit over a wider threshold span, while positive net clinical benefit for PhA was restricted to low-risk thresholds.

## 1 Introduction

Chronic obstructive pulmonary disease (COPD) is a common, complex and heterogeneous disease, and is associated with significant incidence and mortality rates globally (1). In 2015, COPD accounted for an estimated 3.2 million deaths worldwide and is expected to become the third leading cause of death by 2030 (2, 3). The global COPD prevalence rate is estimated to be 4.7% and the overall COPD prevalence rate in Asia is 6.2%. The prevalence rates in Japan and China are 8.5% and 13.6%, respectively. COPD has impacted both developed and developing countries worldwide and is an important global public health issue (4). COPD patients, especially those with severe disease, have substantial medical and economic burdens. Most patients with COPD, especially those with severe conditions, often show significant weight loss and malnutrition, a condition known as pulmonary cachexia. Among COPD patients, 30% to 60% of COPD patients suffer from malnutrition, 20% to 40% have reduced muscle mass, and 10% to 25% have sarcopenia (5). Therefore, it is important to implement preventive measures and effectively manage this condition.

Early screening for malnutrition and timely intervention can help improve the quality of life and disease prognosis for COPD patients. Previous studies have reported (6, 7) that body mass index (BMI) cannot reliably identify malnutrition in COPD patients (8). BMI cannot accurately determine whether increase in adipose tissue or muscle wasting is the cause of obesity in patients. Muscle loss may be offset by increased fat, resulting in an unchanged BMI despite altered body composition (9, 10). The limitations of BMI in evaluating malnutrition were confirmed by another related study, which assessed body composition among COPD patients (11). During a disease state, changes in body composition often occur earlier than the onset of clinical symptoms. Therefore, simple and cost-effective tools are needed to screen for nutritional status. Currently, methods such as dual-energy X-ray absorptiometry are most effective and commonly used in clinical practice. However, they are associated with high costs and cannot be implemented widely (12). In recent years, bioelectrical impedance analysis (BIA) has been widely used for human body composition assessment and evaluate nutritional status because of its economic, portable, non-invasive, and simple operation features (13, 14).

The decline in nutritional status is a common feature in both COPD and aging. Insufficient energy intake can lead to a negative energy balance in the body. This decreases body weight and significant loss of skeletal muscle mass. In clinical applications, PhA measured by BIA not only estimates the muscle ratio of the body, but also serves as an indicator reflecting cell quality and integrity of the cell membrane (14). Current research indicates that PhA effectively assesses nutritional risk and mortality in elderly hospitalized patients (average age of 81.4 years) (15) and in patients with chronic conditions such as cancer (16), type 2 diabetes (17), and those who have undergone surgical procedures (18). The geriatric nutrition risk index (GNRI) is calculated based on serum albumin and body weight and is regarded as an economical and effective indicator for assessing nutritional status. Previous studies have reported (19, 20) that compared to patients with low nutritional risk, those with high nutritional risk among elderly hospitalized patients have longer hospital stays and more significant weight loss during hospitalization. Therefore, GNRI may be an effective screening tool for diagnosing malnutrition, sarcopenia, and physical functional decline.

Compared with other biological markers and complex nutritional assessment methods, routine hospital admission indicators such as blood routine, albumin, and body weight are easily obtainable. Moreover, BIA is not only convenient but also non-invasive. Therefore, it is more suitable for bedside use in hospitals and grassroot applications. Clinically, GNRI can be calculated based on the above basic indicators, and the PhA can be obtained by BIA. This enables preliminary assessment of the patient’s inflammatory level and nutritional status, thereby enabling prediction of the risk of malnutrition and can be used to guide intervention strategies. This study aims to explore and compare the discriminative ability of PhA and GNRI tools in identifying malnutrition in patients with COPD based on the GLIM criterion to provide a reference basis for clinical diagnosis of malnutrition and guide intervention.

## 2 Materials and methods

### 2.1 Design

**Figure 1:**
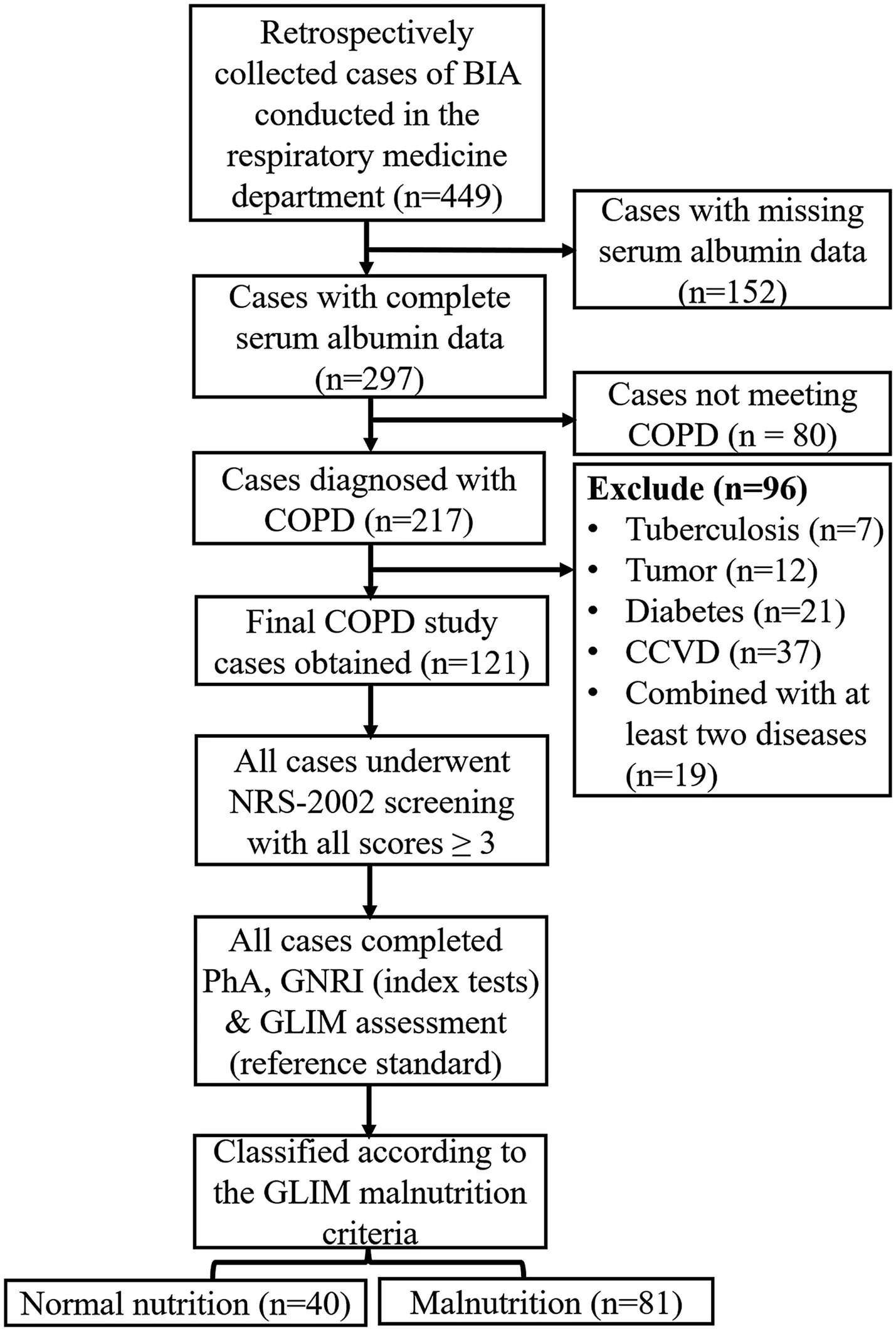
Flowchart illustrating participant screening and enrollment of COPD study cases. GNRI and BIA-based PhA were treated as index tests, with GLIM criteria as the reference standard. BIA, Bioelectrical impedance analysis; CCVD, Cardio-cerebrovascular diseases; GLIM, global leadership initiative on malnutrition.

The clinical data of patients diagnosed with COPD at the Department of Respiratory and Critical Care Medicine of the First Affiliated Hospital of Henan University between 2018 and 2024 was retrospectively collected and systematically organized. The flowchart of patient selection strategy is shown in Figure 1. This study initially limited the screening population to patients who had undergone bioelectrical impedance analysis (BIA) in the Department of Respiratory and Critical Care Medicine between 2018 and 2024. Subsequently, we only enrolled patients with complete biochemical data, including serum albumin measurements required for Geriatric Nutritional Risk Index (GNRI) calculation. Diagnoses of chronic obstructive pulmonary disease (COPD) were identified from hospital medical charts covering the period 2018–2024. Patients complicated with severe systemic or wasting diseases (e.g., active tuberculosis, malignant tumors, severe hepatic and renal dysfunction, diabetes mellitus, cardiovascular and cerebrovascular diseases) were excluded. All patients ultimately enrolled in the analysis were stratified for malnutrition status per the Global Leadership Initiative on Malnutrition (GLIM) criteria. The inclusion criteria for patients were as follows: (1) meeting the diagnostic criteria for COPD documented in inpatient medical records from 2018 to 2024; (2) receiving BIA measurement within our medical institution; (3) having complete biochemical data results containing serum albumin levels required for GNRI calculation; Exclusion criteria were as follows: (1) being unable to cooperate with essential examinations required for the study, such as pulmonary function testing; (2) those with concurrent consumptive diseases such as tuberculosis, tumors, severe liver and kidney dysfunction, diabetes, and cardio-cerebrovascular diseases; (3) lacking complete core data required for analysis, including BIA measurements, serum albumin levels or anthropometric indicators. A total of 449 patients who underwent BIA measurement in the Department of Respiratory and Critical Care Medicine were initially screened. Of these BIA-tested patients, 297 (66.1%) had complete serum albumin data, while 152 patients lacked serum albumin results and were excluded. Subsequent inclusion and exclusion criteria were applied among the 297 patients with albumin data, and 121 hospitalized patients with COPD who completed BIA and had available serum albumin were finally enrolled. This study was approved by the Ethics Committee of the First Affiliated Hospital of Henan University (Approval No. 2025-03-162). All procedures are conducted in accordance with the Declaration of Helsinki guidelines.

### 2.2 Data acquisition

The baseline characteristics, BIA data, and biochemical measurement data collected during hospitalization were retrospectively reviewed. Baseline characteristics included sex, age, height, weight, hospitalization outcomes (length of stay and hospitalization costs), and GLIM malnutrition diagnosis classification. BIA included BMI, phase angle (PhA), and appendicular skeletal muscle index (ASMI). The biochemical measurement data during hospitalization included levels of albumin (ALB), hemoglobin (HGB) and lymphocyte-to-monocyte ratio (LMR), and others. Moreover, BMI (kg/m^2^) = weight (kg) / height (m)^2^.

### 2.3 PhA and ASMI acquisition and nutritional assessment

In this study, professional personnel conducted measurements of hospitalized patients. BIA test was conducted using an InBody S10 bioelectrical impedance analyzer (Biospace, Seoul, South Korea) after overnight fasting or 2 h after a meal, in an environment with a temperature between 23 °C and 25 °C, without any strenuous activity, and removal of metal objects worn on the body. The instrument automatically analyzed and calculated the relevant indicators of body composition, including PhA, ASMI, and others, by measuring impedance values of the human body at different electric current frequencies. In accordance with a previous COPD study (21), a threshold value of 4.25° was used in this study to ensure comparability with the literature and maintain the feasibility of clinical interpretation. PhA > 4.25° was indicative of normal-nourished and PhA ≦ 4.25° indicated malnutrition.

### 2.4 Definition of geriatric nutritional risk index and nutritional assessment

Geriatric nutritional risk index (GNRI) was calculated using serum albumin concentration and body weight as described previously (22). GNRI was calculated as follows: GNRI = 1.489 × albumin (g/L) + 41.7 × weight (kg)/ideal weight (kg). Ideal body weight was calculated separately for men and women using the Lorenz equation (22) as follows: men, Height – 100 − [(Height − 150)/4]; women, Height − 100 − [(Height −150)/2.5]. If the ratio of weight to ideal body weight exceeded or equaled 1.0, it was adjusted to 1. For group stratification, we applied predefined cut-off values from previous studies (23) of patients with COPD to ensure comparability with the literature and maintain clinical interpretability. Specifically, numerous studies (20, 22–26) confirmed that GNRI ≦ 98 serves as the cutoff threshold for identifying patients at nutritional risk. For the purposes of this study, the classification system was simplified to two categories: no risk (GNRI > 98) and at risk (GNRI ≦ 98).

### 2.5 The diagnosis of GLIM malnutrition

The Nutritional risk Screening 2002 tool was used to assess the risk of malnutrition in all patients, with a score of 3 or above indicating a risk of malnutrition (27). Subsequently, the presence of malnutrition in risk patients was diagnosed according to the GLIM criteria. According to the GLIM criteria, at least one of three phenotypic criteria (involuntary weight loss, low BMI, and reduced muscle mass) and one of two etiological criteria (reduced food intake or digestive and absorptive disorders, inflammation or disease burden) need to be met (28). According to previous studies, patients with COPD meet the etiological criteria for inflammation or disease burden (29–31). A consensus report on the GLIM malnutrition criteria explicitly identifies chronic obstructive pulmonary disease (COPD) as a chronic disease burden that fulfils the GLIM etiologic criterion (32). Therefore, the diagnosis of GLIM malnutrition is based on meeting any of the phenotypic criteria. Involuntary weight loss is defined as a weight loss of more than 5% within the past 6 months, or a loss of more than 10% over a period of more than 6 months. A low BMI is defined as less than 18.5 kg/m^2^ for those under 70 years old or less than 20 kg/m^2^ for those over 70 years old (33). According to recommendations from Asian Working Group for Sarcopenia for Asians, reduced muscle mass is defined as ASMI < 7 kg/m^2^ for men and < 5.7 kg/m^2^ for women by BIA (28, 34, 35). In the present cohort, all 121 enrolled patients achieved NRS-2002 ≥ 3. Subsequently, phenotypic assessment for GLIM malnutrition was restricted to low BMI and reduced muscle mass, because standardized data on involuntary weight loss were not consistently documented in our retrospective medical records. All operational definitions for each component of the GLIM criteria were supplemented in the Supplementary material. Detailed measurement methods, cut-off values and assessment procedures for weight loss, reduced food intake, inflammation/disease burden, and low muscle mass are all clearly presented in the Supplementary Tables S1, S2.

### 2.6 Statistical analysis

Continuous variables were presented as mean ± standard deviation, whereas categorical variables were expressed in terms of counts and percentages. To compare the baseline characteristics of the two groups, namely those with malnutrition and those without malnutrition, a Student’s *t*-test was employed for continuous variables, whereas the Chi-Squared test was used for categorical variables. Bootstrap resampling with 1,000 iterations and Hosmer-Lemeshow test verified the reliability and stability of the association between GNRI, PhA, and malnutrition in COPD. ROC curves were used to calculate AUC and screen optimal cutoff values for each diagnostic tool. The DeLong test compared AUC differences across tools. Decision-curve analysis (DCA) was conducted to compare the net clinical benefit of each diagnostic tool. Predicted probabilities for DCA were generated from independent univariate binary logistic regression models, where malnutrition was the outcome, and PhA and GNRI served as the sole diagnostic index in separate models. All statistical tests were two-sided, and *p* < 0.05 was considered statistically significant. All statistical analyses were performed using IBM SPSS software version 27.0, and all the graphs were generated using GraphPad Prism 5.

## 3 Results

### 3.1 Baseline characteristics and hospitalization outcomes

The baseline characteristics of 121 COPD patients completing BIA and with measurable serum albumin are shown in Table 1. The average age of patients diagnosed with COPD was 74.4 ± 10.1 years and included 81.0% males. The LOS was 14.8 ± 9.2 days and the hospitalization costs per day was ¥1450.9 ± 893.9. Furthermore, 81 patients (66.9%) met the criteria for malnutrition as defined by the GLIM. Among the 81 patients who met the GLIM malnutrition criteria, there were 31 cases with low BMI and 80 met the reduced ASMI criteria. Thirty cases met both phenotype criteria. Notably, nearly all malnourished patients in this cohort met the reduced ASMI criterion, with only one patient presenting isolated low BMI.

**Table 1:** Baseline characteristics of COPD patients and hospitalization outcomes.

Variables	Values (*n* = 121)
Age, years	74.4 ± 10.1
Height, cm	166.9 ± 8.6
Weight, kg	62.4 ± 13.1
BMI, kg/m^2^	22.3 ± 4.1
PhA, °	3.9 ± 1.0
ASMI, kg/m^2^	6.5 ± 1.3
Sex, male, *n* (%)	98 (81.0)
LOS, days	14.8 ± 9.2
hospitalization costs per day, ¥	1450.9 ± 893.9
ALB, g/L	36.0 ± 5.0
HGB, g/L	129.4 ± 21.4
LMR	2.29 ± 1.25
GNRI	95.1 ± 11.2
GLIM-defined malnutrition	81 (66.9)

Data were expressed as Mean ± SD or *n* (%). BMI, Body mass index; PhA, Phase angle; ASMI, Appendicular skeletal muscle index; LOS, Length of stay; ALB, Albumin; HGB, Hemoglobin; LMR, Lymphocyte-to-monocyte ratio; GNRI, Geriatric nutritional risk index; GLIM, Global leadership initiative on malnutrition.

### 3.2 Comparison of baseline characteristics and hospitalization outcomes of COPD patients belonging to different sexes

Stratified analyses by sex were performed in the present study and the results are shown in Table 2. Continuous variables were compared using the independent *t*-test, and the chi-square test was used for categorical variables. The results showed significant inter-sex differences in PhA, ASMI, and LMR. Specifically, males showed significantly higher levels of PhA and ASMI but lower levels of LMR relative to females (all *p* < 0.05). The age, BMI, LOS, hospitalization costs per day, albumin, hemoglobin, GNRI and malnutrition were comparable across both sex subgroups (all *p* > 0.05). These findings indicated that PhA, ASMI and LMR were associated with sex, whereas other indicators showed no obvious sex disparities. However, this crude univariate comparison of LOS and hospitalization costs per day did not adjust for potential confounding factors. These observations should be interpreted with caution.

**Table 2:** Comparison of baseline characteristics and hospitalization outcomes for COPD patients belonging to different sexes.

Variables	Male (*n* = 98)	Female (*n* = 23)	*t*/χ^2^ value	*p* value
Age, years	74.0 ± 9.7	76.0 ± 11.5	0.894	0.373
BMI, kg/m^2^	22.4 ± 3.9	22.2 ± 5.1	−0.202	0.840
PhA, °	4.0 ± 1.0	3.4 ± 1.0	−2.598	0.011
ASMI, kg/m^2^	6.8 ± 1.2	5.3 ± 0.9	−5.911	<0.001
LOS, days	15.2 ± 9.5	13.1 ± 7.8	−0.974	0.332
hospitalization costs per day, ¥	1426.8 ± 892.3	1553.4 ± 913.4	0.610	0.543
ALB, g/L	35.9 ± 5.0	36.4 ± 4.9	0.483	0.630
HGB, g/L	129.4 ± 22.0	129.1 ± 18.7	−0.064	0.949
LMR	2.14 ± 1.178	2.92 ± 1.38	2.766	0.007
GNRI	94.9 ± 11.3	96.1 ± 11.1	0.478	0.633
GLIM-defined malnutrition, *n* (%)	64 (65.3)	17 (73.9)	0.624	0.430

Data were expressed as Mean ± SD or *n* (%) by a Student’s *t*-test or Chi-square test. *p* < 0.05 was considered statistically significant. BMI, Body mass index; PhA, Phase angle; ASMI, Appendicular skeletal muscle index; LOS, Length of stay; ALB, Albumin; HGB, Hemoglobin; LMR, Lymphocyte-to-monocyte ratio; GNRI, Geriatric nutritional risk index; GLIM, Global leadership initiative on malnutrition.

### 3.3 Comparison of baseline characteristics and hospitalization outcomes of COPD patients in the malnourished and normal groups diagnosed according to GLIM-criteria

Participants were divided into malnourished and normal groups based on the GLIM criteria, and intergroup comparisons were further analyzed (Table 3). The results showed significant differences in the proportion of patients aged over 75 years, weight, BMI, PhA, ASMI, LOS, HGB, and GNRI between the two groups (all *p* < 0.05), whereas there were no significant differences in hospitalization costs per day, albumin and LMR (all *p* > 0.05). Specifically, the proportion of patients with advanced age (>75 years) in the malnutrition group (56.8%) were significantly higher than those in the normal nutrition group. Furthermore, patients with malnutrition exhibited decreased weight, BMI, PhA, ASMI, hemoglobin, and GNRI, and a longer LOS.

**Table 3:** Baseline characteristics and hospitalization outcomes of COPD patients in the malnourished and normal groups diagnosed according to GLIM-criteria.

Variables	Normal (*n* = 40)	Malnutrition (*n* = 81)	*t*/ χ^2^ value	*p* value
Age over 75, *n* (%)	15 (37.5)	46 (56.8)	3.986	0.046
Weight, kg	73.2 ± 11.5	57.1 ± 10.4	7.739	<0.001
BMI, kg/m^2^	25.8 ± 3.3	20.6 ± 3.3	8.239	<0.001
PhA, °	4.3 ± 1.1	3.7 ± 0.9	2.650	0.010
ASMI, kg/m^2^	7.8 ± 1.0	5.9 ± 0.9	10.580	<0.001
LOS, days	11.8 ± 5.9	16.3 ± 10.2	−3.069	0.003
Hospitalization costs per day, ¥	1532.8 ± 906.3	1410.4 ± 890.6	0.707	0.481
ALB, g/L	36.4 ± 4.0	35.7 ± 5.4	0.676	0.501
HGB, g/L	136.3 ± 21.1	126.0 ± 20.8	2.556	0.012
LMR	2.41 ± 1.37	2.22 ± 1.19	0.763	0.447
GNRI	102.3 ± 8.6	91.6 ± 10.7	5.489	<0.001

Data were expressed as Mean ± SD or *n* (%) by a Student’s *t*-test or Chi-square test. *p* < 0.05 was considered statistically significant. BMI, Body mass index; PhA, Phase angle; ASMI, Appendicular skeletal muscle index; LOS, Length of stay; ALB, Albumin; HGB, Hemoglobin; LMR, Lymphocyte-to-monocyte ratio; GNRI, Geriatric nutritional risk index; GLIM, Global leadership initiative on malnutrition.

### 3.4 Comparison of PhA, ASMI, and LMR in COPD patients in the malnourished and normal groups stratified by sex subgroups

As shown in Table 2, sex was associated with PhA, ASMI, and LMR measurements. Therefore, sex-stratified comparisons of PhA, ASMI, and LMR were performed between malnourished and normal COPD patients. When stratified by sex (Table 4), the results of subgroup analysis for ASMI (all *p* < 0.001) and LMR (all *p* > 0.05) were consistent with those for the overall cohort. However, in male patients, PhA was significantly lower in the malnourished group compared to the normal group (*p* = 0.006). In females, no significant difference in PhA was observed between the two groups (*p* > 0.05).

**Table 4:** Comparison of PhA, ASMI, and LMR for COPD patients in the malnourished and normal groups stratified by sex subgroups.

Variables	Male (*n* = 98)			Female (*n* = 23)		
	Normal (*n* = 34)	Malnutrition (*n* = 64)	*p* value	Normal (*n* = 6)	Malnutrition (*n* = 17)	*p* value
PhA, °	4.4 ± 1.1	3.8 ± 0.9	0.006	3.6 ± 1.3	3.4 ± 0.9	0.631
ASMI, kg/m^2^	8.0 ± 0.9	6.2 ± 0.7	<0.001	6.5 ± 0.2	4.8 ± 0.6	<0.001
LMR	2.26 ± 1.39	2.07 ± 1.05	0.521	3.24 ± 0.97	2.80 ± 1.51	0.444

Data were expressed as Mean ± SD by a Student’s *t*-test. *p* < 0.05 was considered statistically significant. Phase angle, PhA; Appendicular skeletal muscle index, ASMI; Lymphocyte-to-monocyte ratio, LMR.

### 3.5 Comparison of baseline characteristics and hospitalization outcomes for COPD patients in the malnourished and normal groups diagnosed by PhA and GNRI

We evaluated their population association characteristics through baseline characteristic and hospitalization outcome comparisons. Previously established threshold of PhA≦4.25° (malnutrition group) and GNRI≦98 (malnutrition group) for binary classification analysis was used. Table 5 showed the observed variations in baseline characteristics and classical nutritional markers between normal and malnutrition groups, which were categorized according to the PhA and GNRI (PhA≦4.25°, GNRI≦98) nutritional screening tools. The results showed that the prevalence of malnutrition in patients with COPD was 58.7% (71/121) based on PhA screening, and 60.3% (73/121) based on GNRI. In comparison with the normal group, the malnutritional group identified by GNRI and PhA exhibited significantly higher proportion of patients with age over 75 and lower weight, ASMI, albumin, hemoglobin and LMR (all *p* < 0.05). Malnourishment identified by PhA alone was associated with significantly higher hospitalization costs per day (*p* < 0.05). In contrast, malnourishment identified by GNRI alone was associated with significantly lower BMI (*p* < 0.001). Under stratification of the two tools, LOS only showed a numerical trend of prolongation, without reaching statistical significance.

**Table 5:** Comparison of baseline characteristics and hospitalization outcomes for COPD patients in the malnourished and normal groups diagnosed by PhA and GNRI.

Variables	PhA			GNRI		
	Normal (*n* = 50)	Malnutrition (*n* = 71)	*p* value	Normal (*n* = 48)	Malnutrition (*n* = 73)	*p* value
Age over 75, *n* (%)	11 (22.0)	50 (70.4)	0.019	18 (37.5)	43 (58.9)	0.021
Weight, kg	66.8 ± 12.3	59.3 ± 12.9	0.002	71.5 ± 11.5	56.4 ± 10.5	<0.001
BMI, kg/m^2^	23.0 ± 3.9	21.9 ± 4.4	0.150	25.5 ± 3.2	20.2 ± 3.16	<0.001
ASMI, kg/m^2^	6.9 ± 0.9	6.2 ± 1.4	0.001	7.0 ± 1.0	6.2 ± 1.3	<0.001
LOS, days	13.1 ± 7.1	16.1 ± 10.3	0.061	13.3 ± 6.7	15.8 ± 10.5	0.099
hospitalization costs per day, ¥	1190.2 ± 590.4	1634.5 ± 1021.3	0.003	1302.6 ± 580.7	1548.4 ± 1042.8	0.100
ALB, g/L	38.4 ± 4.1	34.3 ± 4.9	<0.001	39.4 ± 3.8	33.7 ± 4.4	<0.001
HGB, g/L	136.3 ± 18.6	124.5 ± 22.0	0.003	135.9 ± 20.9	125.1 ± 20.7	0.006
LMR	2.74 ± 1.29	1.96 ± 1.13	<0.001	2.64 ± 1.35	2.05 ± 1.14	0.011

Data were expressed as Mean ± SD or *n* (%) by a Student’s *t*-test or Chi-square test. *p* < 0.05 was considered statistically significant. BMI, Body mass index; PhA, Phase angle; ASMI, Appendicular skeletal muscle index; LOS, Length of stay; ALB, Albumin; HGB, Hemoglobin; LMR, Lymphocyte-to-monocyte ratio; GNRI, Geriatric nutritional risk index.

### 3.6 Multivariate binary logistic regression analysis to identify correlates for COPD combined with malnutrition

**Figure 2:**
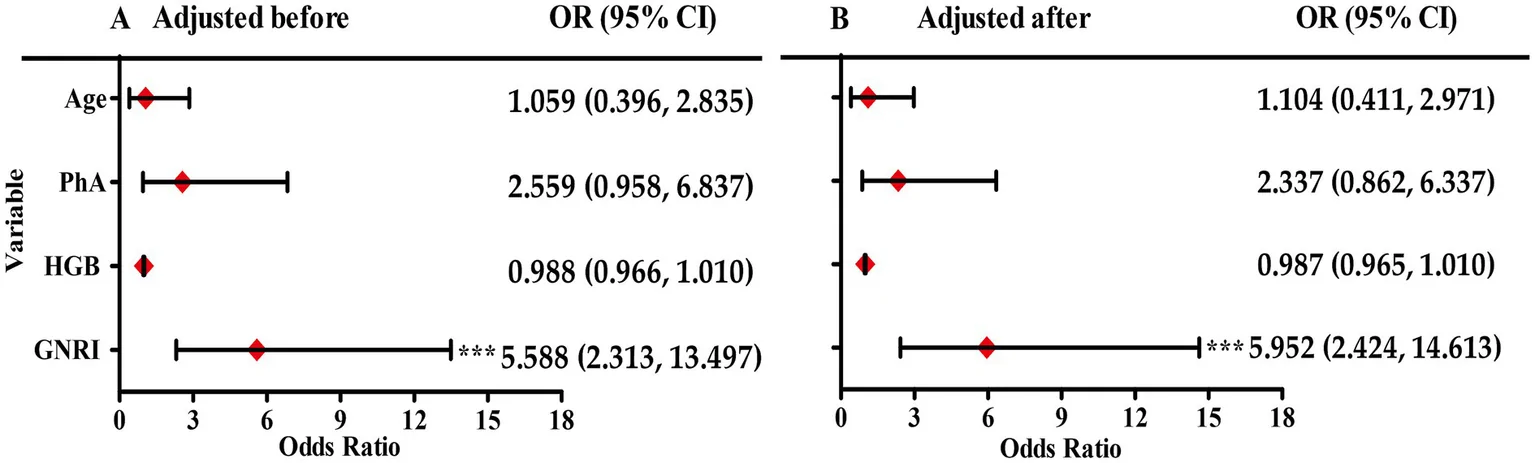
Forest plot of multivariate binary logistic regression analyses for COPD combined with malnutrition **(A)** before and **(B)** after adjustment. PhA, Phase angle; GNRI, geriatric nutritional risk index; HGB, hemoglobin.

To evaluate the independent association of PhA and GNRI with malnutrition in COPD patients and lay a foundation for assessing their individual and combined diagnostic performance, we performed multivariable binary logistic regression analysis. Bootstrap resampling with 1,000 replicates was performed for robustness testing. For the multivariate analysis, COPD combined with malnutrition was used as the binary dependent variable (0 = normal, 1 = malnutrition) and excluded BMI and ASMI indicators related to GLIM diagnosis. The following indicators with statistically significant differences in the single-factor analysis were included in the multivariate regression analyses: age (≦75 years = 0, > 75 years = 1), PhA (>4.25° = 0, ≦4.25° = 1), HGB, and GNRI (>98 = 0, ≦98 = 1). The multivariate binary logistic regression was used to identify factors associated malnutrition in COPD patients. After adjustment for confounding factors across independent variables, results showed that GNRI ≤98 (OR = 5.588, 95% CI: 2.313 ~ 13.497, *p* < 0.001) was significantly independently associated with malnutrition in patients with COPD. Sex was further adjusted as covariates in the multivariable regression analysis. This further increased the association strength GNRI ≤98 (OR = 5.952, 95% CI: 2.424 ~ 14.613, *p* < 0.001) with malnutrition. While PhA showed no significant association regardless of gender adjustment (all *p* > 0.05) (Table 6). Bootstrap resampling with 1,000 test further confirmed the stable and reliable independent association between GNRI and malnutrition. Hosmer-Lemeshow goodness-of-fit test (χ^2^ = 6.606, *p* = 0.580) also verified the reliability of the correlation results. Forest plot of multivariate binary logistic regression analyses for COPD combined with malnutrition was shown in Figure 2. The results of the multivariate binary logistic regression analysis of COPD corrected for malnutrition using 1,000 bootstrap resamples were shown in Supplementary Table S4. Owing to its retrospective design, this study only presents correlational results without causal inference, given unclear variable timing.

**Table 6:** The results of multivariate binary logistic regression analyses for COPD combined with malnutrition.

Variable	Adjusted before		Adjusted after	
	OR (95% CI)	*p* value	OR (95% CI)	*p* value
Age (Ref: ≦75 years)	1.059 (0.396, 2.835)	0.909	1.104 (0.411, 2.971)	0.844
PhA (Ref: > 4.25°)	2.559 (0.958, 6.837)	0.061	2.337 (0.862, 6.337)	0.095
HGB, g/L	0.988 (0.966, 1.010)	0.275	0.987 (0.965, 1.010)	0.269
GNRI (Ref: > 98)	5.588 (2.313, 13.497)	<0.001	5.952 (2.424, 14.613)	<0.001

Sex was adjusted as covariates in the multivariable analysis. Phase angle, PhA; Hemoglobin, HGB; Geriatric nutritional risk index, GNRI; Odds ratio, OR; Confidence interval, CI.

### 3.7 Diagnostic efficacy and net clinical benefit evaluation of PhA and GNRI for malnutrition based on the GLIM-criteria

**Figure 3:**
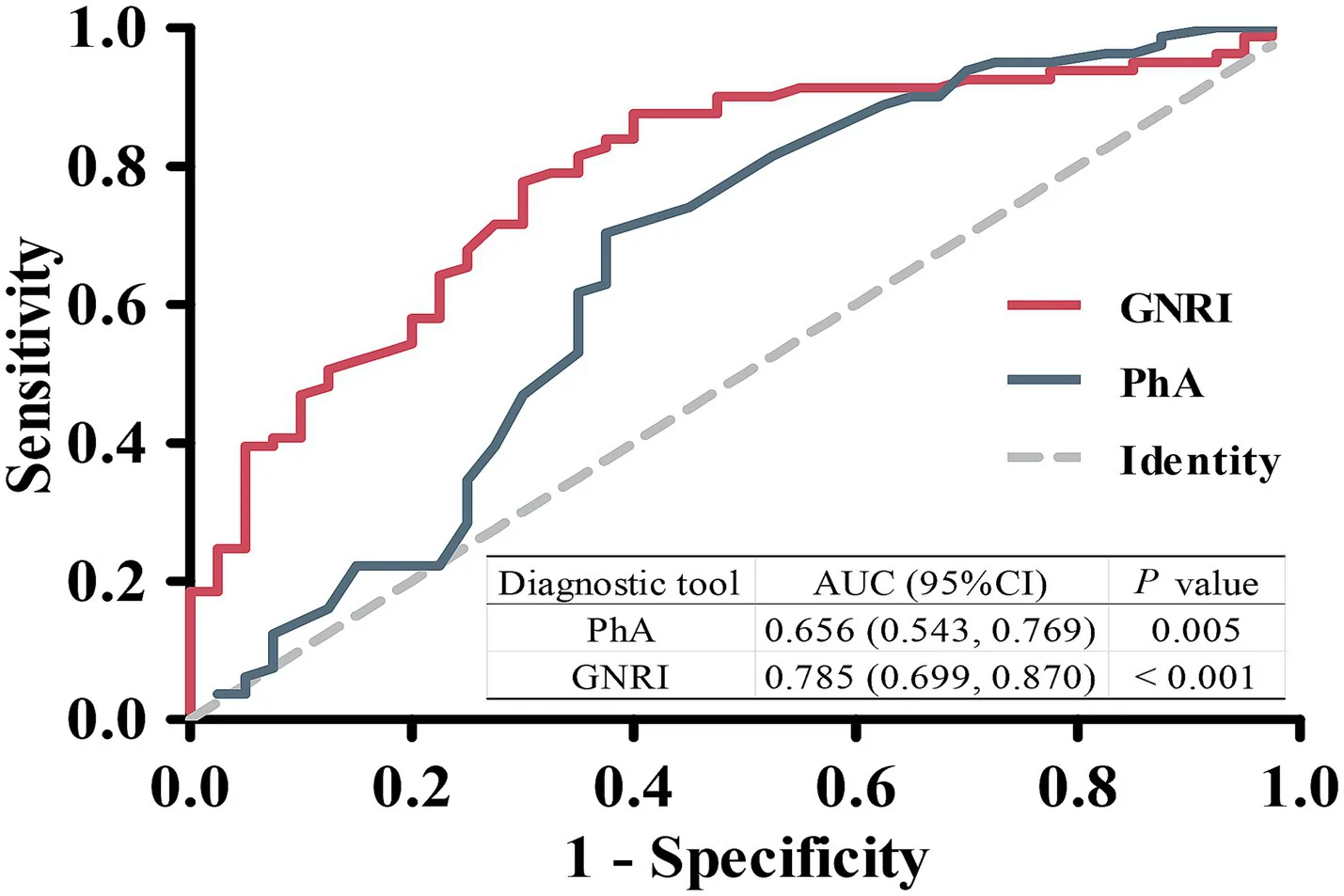
ROC curves for GNRI and PhA. PhA, Phase angle; GNRI, geriatric nutritional risk index.

As shown in Table 7 and Figure 3, PhA and GNRI employed predefined cutoff values of 4.25 and 98 using the GLIM criteria as a reference, respectively. PhA demonstrated a sensitivity of 0.691, specificity of 0.625, positive predictive value of 0.789, negative predictive value of 0.500, accuracy of 0.669, positive likelihood ratio of 1.844, negative likelihood ratio of 0.494 and K value of 0.298. Sensitivity of 0.753, specificity of 0.700, positive predictive value of 0.836, negative predictive value of 0.583, accuracy of 0.736, positive likelihood ratio of 2.510, negative likelihood ratio of 0.353 and K value of 0.431were observed for GNRI. ROC curve analysis revealed AUC values of 0.656 (95% CI: 0.543 ~ 0.769) and 0.785 (95% CI: 0.699 ~ 0.870) for PhA and GNRI, respectively. The DeLong test confirmed that this difference in the AUCs of GNRI and PhA was statistically significant (*p* = 0.035, Supplementary Table S5). Based on the principle of maximizing the Youden index, this study determined the optimal cutoff values for PhA and GNRI to be 4.35 and 98.9, respectively. As illustrated in Supplementary Figure S1, decision-curve analysis revealed that divergent net clinical benefit patterns for GNRI and PhA across risk thresholds. At low thresholds, the net clinical benefit values of GNRI and PhA were close to each other. As the threshold probability increased, the net clinical benefit of PhA rapidly decreased and turned negative. While in a larger threshold range, the net clinical benefit of GNRI remained positive continuously.

**Table 7:** The results of PhA and GNRI for the diagnosis of malnutrition based on the GLIM diagnostic criteria.

Variables	PhA	GNRI
AUC	0.656	0.785
95% CI	0.543 ~ 0.769	0.699 ~ 0.870
Cutoff value	4.35	98.9
Sensitivity	0.691	0.753
Specificity	0.625	0.700
PPV	0.789	0.836
NPV	0.500	0.583
Accuracy	0.669	0.736
LR+	1.844	2.510
LR−	0.494	0.353
K value	0.298	0.431
*p* value	0.005	<0.001

PhA, Phase angle; GNRI, Geriatric nutritional risk index; AUC, Area under curve; PPV, Positive predictive value; NPV, Negative predictive value; LR+, Positive likelihood ratio; LR−, Negative likelihood ratio.

## 4 Discussion

COPD is characterized by chronic and progressive airflow limitation that significantly affects high energy metabolism. Extrapulmonary complications such as skeletal muscle atrophy and malnutrition also significant worsen the clinical prognosis of patients (36). Previous studies have demonstrated that 30 to 60% of patients with COPD are affected by malnutrition (5). The proportion of COPD patients complicated with malnutrition in this study was 66.9%. This figure is slightly higher than the range reported in existing literature. The difference may come from our study population. All participants were COPD inpatients who underwent BIA measurement and possessed valid serum albumin data were ultimately included. Many suffered from acute exacerbations, advanced age, long-term bed rest and poor appetite. Stable outpatients face lower risks of nutritional damage (37). This partly explains the higher malnutrition proportion in our cohort. Malnutrition status deteriorates with disease progression, leading to impaired immune function, which in turn causes structural and functional disorders of the respiratory and skeletal muscles and further exacerbates the disease condition. A previous study (38) showed that the 1-year mortality and hospitalization rates of COPD patients with malnutrition were significantly higher than those without malnutrition. Hospital stays of COPD patients with malnutrition was twice than those for non-malnourished patients and their treatment cost almost doubled. Therefore, malnutrition in COPD patients is related to the patients’ quality of life and the increase of economic burden on individuals and society (22, 39, 40). Our current results showed that the length of stay (LOS) was significantly higher for malnourished COPD patients than for the non-malnourished patients. This intergroup difference is consistent with findings from previous clinical observations. It should be noted that the group differences in LOS derived from univariate analysis merely describe observational patterns without controlling for relevant confounders. Further multivariable analyses adjusting for clinical covariates are required to clarify the independent association between malnutrition and hospitalization outcome among COPD populations. Zhang et al. (22) demonstrated that malnutrition was associated with significant increase in the risks of LOS > 7 days among COPD patients. A large-scale study of over 1.4 million hospitalized COPD patients (41) demonstrated that those with pulmonary cachexia (severe malnutrition) had a significantly longer mean LOS (5.2 days vs. 3.8 days). The LOS increased by approximately 36.8%. These research results all demonstrate the correlation between high risk of malnutrition and additional hospitalization burden. This study is a retrospective observational cohort study. It is difficult to clarify the temporal relationship between the variables and to eliminate the reverse causality bias. The current association cannot distinguish whether malnutrition prolongs the hospital stay of patients, or whether prolonged hospitalization leads to nutritional impairment. The causal direction between these two remains unclear. This requires further prospective research to provide supporting evidence of causal relationship. Although malnutrition is a critical clinical issue in patients with COPD, it is frequently overlooked in routine clinical management. Approximately 20% of COPD patients suffer from weight loss accompanied by protein-calorie malnutrition. Malnutrition in COPD patients exacerbates respiratory muscle dysfunction, aggravates disease severity, and accelerates the progression of physical disability. Moreover, weight loss, low body mass, and malnutrition are also associated with an increased mortality risk in this patient population (42). The body weight, BMI, and ASMI in the malnutrition group were all lower than those in the normal nutrition group in this study. This also suggested that malnutrition was accompanied by a low BMI, weight loss, decrease in muscle mass, muscle atrophy, and even sarcopenia (43). COPD is an inflammatory condition (44), and tend to have this ongoing, low-level inflammation throughout their body (45). This keeps signaling pathways active that break down muscle protein, so their muscle tissue gradually wastes away. Per the 2024 ESPEN guidelines (34), CRP serves as a sensitive biomarker for systemic inflammation, with levels above 3 mg/L indicating an inflammatory state-another qualifying condition under the GLIM etiologic domain. Among the 121 enrolled COPD patients included in our study, 70.2% exhibited elevated CRP, demonstrating a high prevalence of systemic inflammation in this population. This inflammation will interact with other COPD issues, such as long-term hypoxemia, increased respiratory work, and limited mobility. All of these can lead to muscle weakness due to prolonged inactivity (46). If energy intake is insufficient and protein nutrition is deficient at the same time, a vicious cycle of malnutrition, muscle breakdown and deteriorating respiratory function will occur. This pathological mechanism ultimately manifests as a simultaneous decrease in weight, BMI and ASMI (39, 47), etc. It is highly consistent with the observed reduction in muscle mass in this study. The proportion of patients ≥75 years was significantly higher in the malnutrition group (56.8%) than in the normal nutrition group (37.5%). This indicated a statistically significant association between older age and malnutrition of COPD patients. The age distribution characteristics of this study population were consistent with the conclusions of similar studies in recent years. Kang MC, et al. observed males of ≥70 years showed lower muscle strength whereas females showed lower fat-free mass index (48). A cohort study involving 2,824 elderly COPD individuals revealed that patients in the malnutrition risk group were characterized by older age (49). Elderly patients undergo age-related declines in digestion and nutrient absorption. They also frequently lose their appetite. COPD further creates a hypermetabolic burden and restricts daily movement. All these conditions overlap with inadequate food intake, which may explain the higher rates of malnutrition seen in this older population (50).

Malnutrition is defined as a nutritional deficiency, typically because of insufficient caloric and protein intake, and impairs the body’s structural integrity and physiological functions (23). In COPD patients, increased energy expenditure due to labored breathing may lead to malnutrition. Moreover, a spectrum of humoral mediators, ranging from inflammatory cytokines, adipokines, and various endocrine hormones, are implicated as plausible etiological contributors for the development of malnutrition in patients with COPD (51). Furthermore, malnutrition may be associated with reduced physical activity in patients with COPD or with decreased appetite due to depressive tendencies (52). Malnutrition may weaken the patient’s immune defense and accelerate the progression of the disease. Moreover, it can lead to a decline in skeletal muscle mass and function, reduce the quality and thickness of the diaphragm and ultimately result in respiratory failure. Therefore, it is of utmost importance to promptly address the nutritional status and prevent adverse outcomes associated with malnutrition (53). Although the GLIM malnutrition diagnostic criteria released in 2018 have been widely accepted, the clinical implementation was considered insufficient because of the complexity of the steps involved and the ambiguity of factors such as definitions of inflammation or disease burden. This study aims to evaluate the effectiveness of GNRI and PhA in diagnosing malnutrition (as defined by GLIM) in patients with COPD to detect malnutrition more accurately so that nutritional interventions can be implemented as early as possible.

As a specialized nutrition-related risk index, the geriatric nutritional risk index (GNRI) is used to classify elderly patients based on their morbidity and mortality risks associated with pathologies that are commonly complicated by malnutrition. The GNRI is a more reliable prognostic indicator of morbidity and mortality in hospitalized elderly patients than are indices using albumin or BMI alone. The systematic implementation of GNRI enables clinicians to identify patients who are candidates for targeted nutritional intervention (54). GNRI demonstrates superior prognostic prediction performance compared to BMI and ALB levels alone. Its role has been evaluated in recent studies (20, 25) across various cancer types, including lung cancer, head and neck cancer (26), as well as prostate cancer and gastrointestinal cancers. In elderly patients undergoing surgical interventions, accumulating evidence suggests that GNRI (55, 56) can effectively predict a spectrum of adverse postoperative outcomes, including infectious complications, impaired rehabilitation progress, and extended LOS. Under stratification by GNRI, the malnourished group had an average LOS of 15.8 days and an average hospitalization costs per day of 1548.4 yuan in our work. It showed similarity to the study mentioned above. Compared to the normal group, this showed only a slight upward trend, with no statistical difference between the groups. Further research was still needed to prove this. However, the diagnostic value of GNRI in COPD patients with malnutrition remains unclear. Our research findings demonstrated that patients with malnutrition exhibit significantly lower GNRI compared to those with normal nutrition. This suggested that poorer nutritional status of COPD patients was associated with lower GNRI values. The results obtained in this study were in accordance with earlier investigations (26). The GNRI stratification results showed that there were statistically significant differences between groups for the variables of elderly population proportion, ASMI, ALB, HGB, and LMR. Among them, the proportion of elderly population in the malnourished group with GNRI ≦ 98 was significantly higher. The age distribution characteristics are consistent with previous research findings. Huang et al. (57) reported that the mean age of the group with GNRI ≦ 98 was significantly higher. Through GNRI-stratified malnutrition groups, weight and BMI were overall lower. LOS only showed a numerical trend of prolongation, without reaching statistical significance. Multiple studies (57, 58) confirmed that the BMI and weight in the GNRI≦98 group were significantly lower than those in the GNRI >98 group. This pattern was completely consistent with the baseline distribution of this study. The malnourished group showed significantly lower levels of ASMI, ALB, HGB, and LMR compared to the normal group. A retrospective cohort study (22) verified that individuals with a low GNRI often presented with muscle loss, low albumin levels, and a combined state of chronic inflammation, which was in correspondence with the trend of simultaneous decline in multiple indicators observed in this study.

Previous reports have suggested that the PhA is a measurement of the cell’s resistivity, reflecting the integrity of the cell membrane and the overall health of the cell. Healthy cells have a higher impedance and PhA compared to cells with poor nutrition (59). As a sensitive indicator of malnutrition, the PhA can be used to detect clinical changes in nutritional status related to body composition alterations caused by muscle loss or fluid overload. The magnitude of the PhA values is less affected by water content. When patients have edema, the changes in muscle-related indicators do not represent the true situation. At this time, the PhA and edema index can be regarded as more accurate assessment indicators. The PhA has a significant correlation with traditional nutritional screening tools (such as MUST, SGN) and various nutritional risk markers (60). Lower PhA is associated with a deterioration in patient’s prognosis, whereas higher PhA is associated with higher survival rate. Therefore, nutritional status of the patient can be non-invasively evaluated by monitoring their PhA (61). Our results demonstrate that PhA of participants with malnutrition is significantly lower compared to those with normal nutrition. This suggests that the poorer nutritional status of COPD patients corresponds to lower PhA values. This finding is consistent with previously reported results. However, in male patients, PhA was significantly lower in the malnourished group compared to the normal group. In females, no significant difference in PhA was observed between the two groups. The negative findings observed in female patients may simply reflect insufficient statistical power rather than the absence of an association. The two groups of malnutrition (PhA≦4.25°) and normal nutrition (PhA > 4.25°) classified by PhA tools all showed a trend of increasing proportion of elderly population, as well as decreased levels of ASMI, ALB, HGB, and LMR. The percent of 70.4 were over 75 years old in the malnutrition group. This was significantly higher than the 22.0% in the normal nutrition group (*p* = 0.019), indicating a higher proportion of elderly in the malnutrition group. Kajiyama et al. (62) found that older participants showed lower PhA values. The results of the correlation analysis indicated that PhA was negatively correlated with age. Weight and BMI showed no statistical difference in PhA-stratified populations (*p* > 0.05), which was consistent with the previous studies (63). This might be due to a falsely elevated BMI caused by edema in COPD patients. A multi-center prospective cohort from China (*n* = 652) (64) discovered that 36.3% of patients with COPD experienced edema. This also indirectly proves that PhA based on BIA reflects cell membrane integrity and nutritional status. It is not directly constrained by BMI or body fat percentage, and can identify hidden malnutrition populations with normal BMI but experiencing muscle atrophy and impaired cellular function (59). The malnutrition group had a slightly LOS than the normal group without statistical significance (*p* = 0.061). Its average hospitalization costs per day was markedly elevated (1634.5 ¥ vs. 1190.2 ¥, *p* = 0.003) under PhA stratification. Prior research (65) confirmed that a low PhA was associated with chronic inflammation activation and a high incidence of hospital complications. A persistent inflammatory state increased the consumption of medical resources. This also confirmed the result of the present work that low PhA patients had higher average daily hospital costs from a mechanistic perspective.

Multivariate binary logistic regression analysis adjusted for confounding factors showed that GNRI≦ 98 was significantly associated with GLIM malnutrition before and after adjustment (OR = 5.952, 95% CI: 2.424 ~ 14.613, *p* < 0.001), with stable independent correlation. However, PhA only showed a marginal trend related to malnutrition before correction (*p* = 0.061), and the correlation was completely non-statistically significant after correction of all confounders (*p* = 0.095). We further analyzed the diagnostic value of GNRI for malnutrition in COPD patients. The ROC curve analysis determined that the optimal cutoff value for GNRI was 98.9. Chen et al. (24) indicated that GNRI ≤ 98 was the optimal cutoff value for predicting malnutrition in rectal cancer patients and showed a sensitivity of 0.778 and specificity of 0.690. This was broadly consistent with our study results. Meanwhile, the optimal cut-off value of PhA for predicting malnutrition in COPD patients was 4.35°. Murakami et al. (21) reported that the optimal cutoff value for PhA to diagnose malnutrition in COPD patients was 4.25° with a sensitivity of 0.570 and a specificity of 0.820. This was slightly similar to our findings. Fernández-Jiménez et al. (66) focused on the application of PhA as a screening tool for nutritional risk in hospitalized patients.

The AUC of GNRI was 0.785 (95% CI: 0.699 ~ 0.870), indicating a moderate diagnostic level. GNRI yielded sensitivity of 0.753, specificity of 0.700, accuracy of 0.736 and a K value of 0.431. In contrast, the AUC of PhA was 0.656 (95% CI: 0.543 ~ 0.769), with distinct sensitivity (0.691), specificity (0.625), accuracy (0.669) and K value (0.298). Discriminative capacity of PhA shows weak agreement with the GLIM standard. Additionally, GNRI and PhA displayed different positive and negative likelihood ratios. GNRI exhibited a higher positive likelihood ratio and a lower negative likelihood ratio compared with PhA. A comparative diagnostic study on gastric cancer patients also used the GLIM standard as the gold standard (67). The AUC of GNRI was measured at 0.805 and the K value was 0.452. This data was highly similar to the results of this study, indicating that the diagnostic efficacy of GNRI have a moderate agreement with the GLIM standard. It also suggests that this tool may have acceptable clinical consistency with the GLIM standard in different disease contexts. Further research is needed for verification. Mullie et al. (60) reported that the PhA can be used to monitor the clinical changes in nutritional status related to body composition alterations caused by muscle loss or fluid overload. When patients have edema, the changes in muscle-related indicators (such as BMI) are not the true situation. In specific clinical situations, the PhA may be a more accurate assessment indicator. This was consistent with the result of our study where there was no statistical difference in BMI stratified by PhA.

The decision curve analysis (DCA) further illustrated the net clinical benefit characteristics of the two tools from the perspective of clinical intervention benefits (68). When the threshold was low, the benefit levels of the two decisions were similar. As threshold probability increased, the net clinical benefit of PhA declined sharply and soon fell below zero. GNRI maintained a positive net clinical benefit within a wider range of thresholds. These divergent patterns of net clinical benefit implied differences in the suitable risk intervals for clinical application between the two tools. These results indicate that PhA and GNRI are expected to serve as feasible supplementary tools for assessing malnutrition in this COPD patient population. GNRI is multidimensional and reflects the nutritional and inflammatory status of the body (24). PhA reflects cellular function and nutritional status of the body (69). The observed differences in performance may partly relate to clinical features of COPD, including a higher prevalence of fluid overload and edema. These factors may interfere with the capacity of PhA to identify GLIM-defined malnutrition. This hypothesis requires further verification in subsequent investigations. Previous work (60) indicated potential differences between PhA and anthropometric indicators such as BMI, weight, and ASMI under certain clinical circumstances, where fluid retention may limit the reliability of these body mass metrics. Additionally, existing study suggests PhA may display unique characteristics when exploring associations with hospital-related outcomes (such as hospitalization time). But this hypothesis requires further research to verify these preliminary observations.

This study has several limitations. Firstly, it was a single-center retrospective study that cannot establish causality. The small sample size, particularly the limited number of female patients, weakened the statistical power of gender-stratified analyses. Negative results may stem from inadequate sampling rather than a true lack of association. In addition, missing data on COPD severity (including GOLD stage, pulmonary function parameters, and symptom scores) precluded full confounding adjustment, potentially leaving residual bias. Secondly, nutritional assessment had inherent variable overlap and incorporation bias, as PhA, ASMI and GNRI share core GLIM phenotypic indicators. Stratified ROC analyses partially mitigated but could not fully eliminate this bias. Missing data on weight loss and dietary intake may underestimate malnutrition prevalence. Nearly all malnourished patients met the reduced ASMI criterion. Only one patient showed isolated low BMI. This may reflect common muscle wasting in COPD. Interpretation was limited due to incomplete GLIM criteria. The unadjusted LOS comparison only revealed correlations without causal inference due to potential reverse causality and temporal ambiguity. For DCA, predicted probabilities were derived from univariate logistic models. Further research could assess their net clinical benefit adjusted for clinical confounders. Thirdly, the study exhibited obvious selection bias. Patients without valid albumin data were excluded, and the cohort only included patients receiving BIA testing, excluding untested hospitalized COPD individuals. Therefore, our sample is a selectively tested subgroup, restricting the generalizability of the findings. In conclusion, all analyses in this study were exploratory, and the discriminative performance comparison between GNRI and PhA should be interpreted with caution. Future multicenter prospective studies with standardized data collection are needed to validate and extend our results.

## 5 Conclusion

In conclusion, this study shows that both PhA and GNRI tools have discriminative capacity for identifying malnutrition in patients with COPD. PhA shows weak discriminative capacity, and GNRI has moderate good discriminatory ability in this cohort. Both tools may represent a promising adjunctive screening tool, but they exhibit different performance characteristics. The effective threshold ranges of the two tools are different. GNRI demonstrated net clinical benefits within a wider threshold range, while the net clinical benefits of PhA were limited to the low-risk threshold. The two tools provide a useful supplement to existing malnutrition screening tools from the dual perspectives of immune factors and cellular functions. Further research is needed to verify our conclusions.

## Data Availability

The original contributions presented in the study are included in the article/Supplementary material, further inquiries can be directed to the corresponding author.
