# Supplementary material for: Diagnostic value of geriatric nutritional risk index and phase angle for malnutrition in patients with COPD: a retrospective cohort study
Source: Front Nutr. 2026 Aug 19;13:1887430. doi: 10.3389/fnut.2026.1887430 (PMC13533752; doi:10.3389/fnut.2026.1887430)
Supplement: Supplementary file 1 [file Table_1.DOCX]

Supplementary Material

**Supplementary Table S1.** Examples of Recommended Thresholds for Reduced Muscle Mass.^[1, 2]^

| ASMI, (kg/m^2^) ^a^ | Males | Females |
| --- | --- | --- |
| DXA | ＜7 | ＜5.4 |
| BIA | ＜7 | ＜5.7 |

DXA = dual energy x-ray absorptiometry, BIA = bioelectrical impedance analysis

^a^ Recommendations from Asian Working Group for Sarcopenia (AWGS) for Asians

**Supplementary Table S2.** Phenotypic and Etiologic Criteria for the Diagnosis of Malnutrition.^[1]^

| Category | Index | Diagnostic Cutoffs |
| --- | --- | --- |
| Phenotypic Criteria | Weight Loss (%) | >5% weight loss within 6 months, or >10% loss over 6 months |
|  | Low Body Mass Index (kg/m^2^) | Asians:  <70y: BMI<18.5, ≥70y: BMI <20 |
|  | Decreased Muscle Mass ^2^ | Confirmed via validated body composition tests. Mid-arm/calf circumference as alternatives; race-specific thresholds for Asians. Handgrip strength as supportive evidence. |
| Etiologic Criteria ^1^ | Poor Intake/Malabsorption ^3, 4^ | Intake ≤ 50% energy demand for > 1 week, or any reduced intake > 2 weeks, or any chronic gastrointestinal disorders disrupting food uptake and nutrient absorption |
|  | Inflammation ^5, 6, 7^ | Caused by either acute illness/injury ^5, 7^ or long-term chronic disease ^6, 7^ |

^1^ General diagnostic rule: Malnutrition diagnosis needs at least one phenotypic and one etiologic marker.

^2^ Muscle mass assessment: DXA-derived fat-free mass index, BIA, CT or MRI are gold-standard methods. If unavailable, physical anthropometry (mid-arm muscle/calf circumference) works, with Asian-specific cutoffs. Handgrip test supports assessment.

^3^ Gastrointestinal symptoms: Dysphagia, nausea, vomiting, diarrhea, constipation or abdominal pain impair intake/absorption. Judge severity by symptom intensity, frequency and duration.

^4^ Malabsorption: Seen in short bowel syndrome, pancreatic insufficiency, post-bariatric surgery, esophageal stricture, gastroparesis and pseudo-obstruction. Key signs: chronic diarrhea, steatorrhea, high ostomy output. Assess severity via stool fat and fluid loss volume.

^5^ Acute inflammation: Severe inflammation accompanies major infection, burns, trauma or closed head injury; mild-moderate inflammation occurs in other acute conditions.

^6^ Chronic inflammation: Chronic mild/moderate inflammation links to cancer, COPD, heart failure, chronic kidney disease. Temporary mild inflammation does not qualify.

^7^ Lab marker: C-reactive protein (CRP) serves as an auxiliary inflammatory biomarker.

**Supplementary Table S3.** Comparison of Characteristics and Malnutrition Status of COPD Patients Based on CRP Levels: A Sensitivity Analysis

| **Variables** | **CRP ≤ 3 mg/L or CRP deficiency (n=36)** | **CRP > 3 mg/L (n=85)** | ***t*/χ^2^ value** | ***P* value** |
| --- | --- | --- | --- | --- |
| Age, years | 74.444 ± 9.3027 | 74.318 ± 10.4074 | 0.063 | 0.950 |
| Height, cm | 167.444 ± 8.9425 | 166.729 ± 166.729 | 0.414 | 0.679 |
| Weight, kg | 62.167 ± 12.9123 | 62.494 ± 13.3172 | -0.125 | 0.901 |
| BMI, kg/m^2^ | 22.0578 ± 3.68225 | 22.4338 ± 4.28889 | -0.459 | 0.647 |
| Sex, male | 26 (72.2) | 72 (84.7) | 2.560 | 0.110 |
| LOS, days | 14.444 ± 7.1051 | 14.976 ± 10.0261 | -0.289 | 0.773 |
| hospitalization costs per day, ¥ | 1268.4467 ± 659.63849 | 1528.1286 ± 969.46753 | -1.468 | 0.145 |
| GLIM-defined malnutrition | 21 (58.3) | 60 (70.6) | 1.716 | 0.190 |

Notes: Data were expressed as Mean ± SD or n (%). C-reactive protein, CRP; Body mass index, BMI; Length of stay, LOS; Global leadership initiative on malnutrition, GLIM.

**Supplementary Table S4.** The results of multivariate binary logistic regression analyses for COPD combined with malnutrition corrected by 1000 bootstrap resamples

| **Variable** | **Adjusted before** | |  | **Adjusted after** | |
| --- | --- | --- | --- | --- | --- |
|  | **OR (95% CI)** | ***P* value** |  | **OR (95% CI)** | ***P* value** |
| Age (Ref: ≦75 years) | 1.059 (0.360, 3.216) | 0.918 |  | 1.104 (0.354, 3.184) | 0.839 |
| PhA (Ref: > 4.25º) | 2.559 (0.983, 8.298) | 0.053 |  | 2.337 (0.826, 7.925) | 0.095 |
| HGB, g/L | 0.988 (0.958, 1.010) | 0.295 |  | 0.987 (0.960, 1.011) | 0.283 |
| GNRI (Ref: > 98) | 5.588 (2.330, 15.800) | <0.001 |  | 5.952 (2.392, 18.541) | <0.001 |

Notes: Sex was adjusted as covariates in the multivariable analysis. Phase angle, PhA; Length of stay, LOS; Hemoglobin, HGB; Geriatric nutritional risk index, GNRI; Odds ratio, OR; Confidence interval, CI. OR and 95% CI were transformed from bootstrap regression coefficients.

**Supplementary Table S5.** DeLong test for pairwise AUC comparison among PhA, GNRI and combined tool of GNRI-PhA model

| Comparative diagnostic tools | AUC difference | SE | 95% CI | *Z* value | *P* value |
| --- | --- | --- | --- | --- | --- |
| PhA and GNRI | 0.128 | 0.0609 | 0.009 ~ 0.248 | 2.108 | 0.035 |

Notes: Phase angle, PhA; Geriatric nutritional risk index, GNRI; Area under the curve, AUC; Confidence interval, CI.

**Supplementary Figure S1.** Decision curve analysis of the exploratory net clinical benefit of the PhA and GNRI tools under different risk thresholds. Notes: Phase angle, PhA; Geriatric nutritional risk index, GNRI.

**Supplementary Figure S2.** ROC curves for GNRI and PhA stratified by ASMI (A) and BMI (B) respectively. A low BMI is defined as less than 18.5 kg/m^2^ for those under 70 years old or less than 20 kg/m^2^ for those over 70 years old. According to recommendations from Asian Working Group for Sarcopenia for Asians, reduced muscle mass is defined as ASMI < 7 kg/m^2^ for men and < 5.7 kg/m^2^ for women by BIA. Notes: Phase angle, PhA; Appendicular skeletal muscle index, ASMI; Geriatric nutritional risk index, GNRI.

**References**

1. Jensen GL, Cederholm T, Correia M, Gonzalez MC, Fukushima R, Higashiguchi T, et al. **GLIM Criteria for the Diagnosis of Malnutrition: A Consensus Report From the Global Clinical Nutrition Community**. *JPEN J Parenter Enteral Nutr* 2019; 43(1):32-40.

2. Chen LK, Lee WJ, Peng LN, Liu LK, Arai H, Akishita M. **Recent Advances in Sarcopenia Research in Asia: 2016 Update From the Asian Working Group for Sarcopenia**. *J Am Med Dir Assoc* 2016; 17(8):767.e761-767.
